# Liquid Chromatography/Tandem Mass Spectrometry-Based Simultaneous Analysis of 32 Bile Acids in Plasma and Conventional Biomarker-Integrated Diagnostic Screening Model Development for Hepatocellular Carcinoma

**DOI:** 10.3390/metabo14090513

**Published:** 2024-09-23

**Authors:** Minami Yamauchi, Masamitsu Maekawa, Toshihiro Sato, Yu Sato, Masaki Kumondai, Mio Tsuruoka, Jun Inoue, Atsushi Masamune, Nariyasu Mano

**Affiliations:** 1 Graduate School of Pharmaceutical Sciences, Tohoku University, 1-1 Seiryo-machi, Aoba-Ku, Sendai 980-8574, Japanmano@hosp.tohoku.ac.jp (N.M.); 2 Department of Pharmaceutical Sciences, Tohoku University Hospital, 1-1 Seiryo-machi, Aoba-Ku, Sendai 980-8574, Japan; toshihiro.sato@tohoku.ac.jp (T.S.); yu.sato.e7@tohoku.ac.jp (Y.S.); masaki.kumondai.d5@tohoku.ac.jp (M.K.); 3 Advanced Research Center for Innovations in Next-Generation Medicine, Tohoku University, 1-1 Seiryo-machi, Aoba-Ku, Sendai 980-8574, Japan; 4 Division of Gastroenterology, Tohoku University Graduate School of Medicine, 1-1 Seiryo-machi, Aoba-Ku, Sendai 980-8574, Japanjun.inoue.d8@tohoku.ac.jp (J.I.); amasamune@med.tohoku.ac.jp (A.M.)

**Keywords:** biomarkers, hepatocellular carcinoma, bile acids, plasma, LC-MS/MS, screening

## Abstract

Imaging tests, tumor marker (TM) screening, and biochemical tests provide a definitive diagnosis of hepatocellular carcinoma (HCC). However, some patients with HCC may present TM-negative results, warranting a need for developing more sensitive and accurate screening biomarkers. Various diseases exhibit increased blood levels of bile acids, biosynthesized from cholesterol in the liver, and they have been associated with HCC. Herein, we analyzed plasma bile acids using liquid chromatography/tandem mass spectrometry and integrated them with conventional biomarkers to develop a diagnostic screening model for HCC. Plasma samples were obtained from patients diagnosed with chronic hepatitis, hepatic cirrhosis (HC), and HCC. A QTRAP 6500 mass spectrometer and a Nexera liquid chromatograph with a YMC-Triart C18 analytical column were used. The mobile phase A was a 20 mmol/L ammonium formate solution, and mobile phase B was a methanol/acetonitrile mixture (1:1, *v*/*v*) with 20 mmol/L ammonium formate. After determining the concentrations of 32 bile acids, statistical analysis and diagnostic screening model development were performed. Plasma concentrations of bile acids differed between sample groups, with significant differences observed between patients with HC and HCC. By integrating bile acid results with conventional biochemical tests, a potential diagnostic screening model for HCC was successfully developed. Future studies should increase the sample size and analyze the data in detail to verify the diagnostic efficacy of the model.

## 1. Introduction

Liver cancer is the third leading cause of cancer-related death worldwide [1], with hepatocellular carcinoma (HCC) accounting for 75–85% of all liver cancers [1]. HCC mostly develops from hepatic cirrhosis (HC), caused by chronic inflammation following hepatitis virus infection. More than 60% of patients with HCC are diagnosed at a later stage when metastasis has occurred because of difficulty in diagnosis, resulting in a 5-year survival rate of <20% [2,3]. However, if diagnosed at an early stage, the prognosis improves significantly, with the 5-year survival rate exceeding 70% [2,4]. Therefore, early diagnosis and initiation of HCC treatment are crucial to achieve a better prognosis.

HCC is mainly diagnosed using imaging modalities, such as computed tomography and magnetic resonance imaging, when nodular lesions are detected by abdominal ultrasound [5,6,7,8]. Nevertheless, despite ultrasound being the initial diagnostic procedure for HCC, its result analysis is subject to operator dependency and exhibits inherent limitations in detecting small tumors [9,10,11,12]. Concomitant liver diseases or body habitus, such as metabolic dysfunction-associated steatohepatitis (MASH) or obesity, can impair the efficacy of ultrasound imaging, resulting in diminished sensitivity. Additionally, serum tumor marker (TM) tests are performed to complement ultrasound results. Three TMs, namely α-fetoprotein (AFP) [13,14,15,16,17], protein induced by the absence of vitamin K or antagonist-II (PIVKA-II) [16,17,18,19,20], and AFP-L3 [17,21,22], have been identified and measured in HCC surveillance. AFP is a glycoprotein that is normally produced in the liver and gastrointestinal tract during fetal and neonatal development. In patients with HCC, AFP serum levels are increased, and their usefulness in the diagnosis of HCC has been confirmed in a randomized controlled trial. AFP is further divided into three glycoforms, namely AFP-L1, AFP-L2, and AFP-L3, based on their binding affinity to lectins; AFP-L3 is the binding fraction that increases in HCC. Although AFP-L3 levels are correlated with AFP levels, AFP-L3 shows higher specificity than AFP because AFP-L3 is derived from cancer cells. PIVKA-II is an abnormal prothrombin in which γ-carboxylation of glutamic acid residues in the *N*-terminal domain is incomplete from the prothrombin precursor. Although PIVKA-II levels are increased in the serum of patients with HCC, there is no established correlation between PIVKA-II and AFP levels; hence, PIVKA-II could serve as a complementary TM in HCC diagnosis. Although these biomarkers have some utility, their diagnostic performances remain incomplete. Increased AFP levels have been reported to be increased in benign liver diseases, such as HC and chronic hepatitis, which may lead to poor specificity. AFP-L3 shows higher specificity than AFP but has been reported to be undetectable in patients with AFP levels < 20 ng/mL. Whether PIVKA-II is useful for HCC screening remains controversial as evidence remains lacking, especially in Western populations. Moreover, these biomarkers are negative in approximately 30% of patients at an early stage [23,24]. Thus, monitoring AFP during surveillance is treated as an option or not recommended, while PIVKA-II and AFP-L3 are not recommended in clinical guidelines other than Japan [25,26].

Bile acids are amphiphilic molecules synthesized from cholesterol in the liver [27,28,29,30] and are involved in essential physiological functions such as glucose and energy metabolism, cholesterol metabolism, and cellular immunity [31]. In individuals with healthy liver function, approximately 95% of bile acids are produced in enterohepatic circulation, and bile acids in systemic circulation are maintained at low levels [29,32,33,34]. Conversely, in cases of liver disease, the enterohepatic circulation is disrupted, which results in increased levels of bile acids in the blood and urine [35,36,37,38,39]. Increased bile acid levels have been reported in various liver diseases, such as cholestasis, HC, MASH, and even HCC [40,41,42,43,44,45,46].

Recently, bile acids have been reported to be involved in HCC development [41,47,48]. While bile acids play important roles in various physiological functions, they can induce inflammation and reactive oxygen species production, and reduce the apoptosis of DNA-damaged cells [40,49]. Therefore, more detailed relationships between serum bile acid levels and HCC development have been investigated, including whether bile acids can serve as diagnostic or risk classification markers for HCC. Stepien et al. performed untargeted metabolomics and showed strong positive associations between HCC risk and circulating levels of glycine-conjugated cholic acid (GCA) and glycine-conjugated chenodeoxycholic acid (GCDCA) [50]. They further performed targeted metabolomics for bile acids and revealed that the profile of plasma bile acids shifted toward increased proportions of taurine conjugates, along with increased total bile acid concentration several years before HCC diagnosis [51]. Khalil et al. measured 14 bile acids in patients with non-HC, HC, and HCC following hepatitis C infection and in control subjects. Additionally, the serum concentrations of some bile acids, including taurine-conjugated cholic acid (TCA), GCA, and glycine-conjugated ursodeoxycholic acid (GUDCA), were increased in patients with HCC, followed by those with HC, non-HC, and healthy controls [52]. Ressom et al. conducted untargeted metabolomics and quantitative analyses of bile acids in the sera of patients with HCC and CH. The findings revealed a notable downregulation of GCA, glycine-conjugated deoxycholic acid (GDCA), TCA, and taurine-conjugated chenodeoxycholic acid (TCDCA) in patients with HCC compared to those with HC [53]. Serum metabolomics analysis of patients with HCC and HC, and healthy individuals performed by Han et al. revealed that chenodeoxycholic acid (CDCA) and GCA were downregulated in the serum of patients with HCC compared to that of patients with HC [54]. Thus, the profile of bile acids in HCC could be altered from that in other liver diseases, and bile acids could serve as biomarkers. However, whether bile acids change during the development of HCC has been inconsistent in previous reports, and their diagnostic value remains controversial. In addition, studies in the Japanese population are limited.

In this study, we quantified 32 bile acids in the plasma of patients with HCC and other liver diseases using liquid chromatography (LC)-tandem mass spectrometry (MS/MS) to evaluate their diagnostic performance for HCC. Our method is distinguished by the incorporation of bile acids with diverse characteristics, including sulfate and glucuronide conjugates. This approach is promising for the development of highly innovative models. Furthermore, we developed and evaluated a diagnostic screening model for HCC by integrating bile acid levels and conventional biochemical tests.

## 2. Materials and Methods

### 2.1. Chemicals and Reagents

The structures of bile acids are shown in Figure S1. CA, CDCA, and taurine-conjugated lithocholic acid (TLCA) were purchased from Sigma-Aldrich (St. Louis, MO, USA). Ammonium formate, deoxycholic acid (DCA), lithocholic acid (LCA), and methanol were purchased from FUJIFILM Wako Pure Chemical Corporation (Osaka, Japan). Ursodeoxycholic acid (UDCA), GUDCA, TCA, and taurine-conjugated ursodeoxycholic acid (TUDCA) were purchased from Nacalai Tesque Inc. (Kyoto, Japan). LCA-2,2,4,4-[^2^H] (LCA-[^2^H_4_]) was purchased from Cambridge Isotope Laboratories (Tewksbury, MA, USA). GCA, GCDCA, GDCA, glycine-conjugated lithocholic acid (GLCA), TCDCA, taurine-conjugated deoxycholic acid (TDCA), CDCA 3-sulfate (3S), DCA 3S, LCA 3S, GCA 3S, GCDCA 3S, GDCA 3S, GUDCA 3S, GLCA 3S, TCA 3S, TCDCA 3S, TDCA 3S, TUDCA 3S, TLCA 3S, cholic acid (CA) 3-glucuronide (3GlcA), CDCA 3GlcA, DCA 3GlcA, UDCA 3GlcA, LCA 3GluA [55], CA-3,7,12-[^18^O], DCA-2,2,4,4-[^2^H], GCA-3,7-[^18^O], GCDCA-3,7-[^18^O,^2^H] (GCDCA-[^18^O_2_,^2^H_2_]), GLCA-3-[^18^O,^2^H], TDCA-3,12-[^18^O,^2^H], TLCA-3-[^18^O,^2^H], 17β-estradiol-2,4,16,16-[^2^H] 3-sulfate, and 3β-sulfooxy-7β-hydroxy-23-*nor*-5-cholen-oic acid [56], previously synthesized in our laboratory, were used. Ethanol was purchased from the Japan Alcohol Trading Co., Ltd. (Tokyo, Japan). Acetonitrile was purchased from Kanto Chemical Co., Inc. (Tokyo, Japan). Ultrapure water was purchased from Puric-α (Organo Corporation, Tokyo, Japan).

### 2.2. Plasma Samples

This study was conducted in accordance with the protocol approved by the Ethics Committee of the Graduate School of Medicine, Tohoku University (approval number: 2020-1-732). The inclusion criteria were (1) patients with liver diseases such as chronic hepatitis, HC, and HCC; (2) 20 years or older. Patients whose clinical data were unavailable were excluded. We published the online research information and established the opt-out option because the samples were previously collected. According to these criteria, plasma samples from patients with chronic hepatitis (*n* = 20), HC (*n* = 20), and HCC (*n* = 39) were included. These samples were collected at Tohoku University Hospital between 2011 and 2019. Patients with HCC were divided into two groups based on their TM status: TM-negative (TM−) and TM-positive (TM+). Patients who met all of the following criteria were classified as TM− (*n* = 19): AFP ≤ 10 ng/mL, PIVKA-II ≤ 40 mAU/mL, and AFP-L3 < 10%. The TM+ HCC group comprised individuals who did not meet at least one of the specified criteria (*n* = 20).

The previously measured clinical test values were used in this study. Biomarkers of liver injury included gamma-glutamyltransferase (GGT), aspartate aminotransferase (AST), and alanine aminotransferase (ALT). Biomarkers of liver function included serum albumin (ALB) and cholinesterase (ChE) Serum creatinine (SCr) was used as a biomarker of renal function. AFP, PIVKA-II, and AFP-L3 were used as TMs for HCC.

### 2.3. Plasma Bile Acid Analysis by LC-MS/MS

For deproteinization, 50 µL of the internal standard (IS) mixture and 400 µL of acetonitrile were added to each plasma sample (50 µL) and vortexed. Subsequently, centrifugation was performed at 15,000× *g* for 5 min at 4 °C, after which 450 µL of the supernatant was transferred to another tube and dried. The residue was dissolved in 50 µL of a water/methanol solution (1:1, *v*/*v*), and 10 µL of aliquots were measured.

A Nexera ultra-high-performance liquid chromatography system (Shimadzu Corporation, Kyoto, Japan) equipped with a QTRAP6500 quadrupole linear ion trap hybrid tandem mass spectrometer and an electrospray ionization probe (SCIEX, Framingham, MA, USA) were used. The LC-MS/MS conditions were based on a previously established method in our laboratory [45]. For LC, 20 mmol/L aqueous ammonium formate and a mixture of methanol/acetonitrile (1:1, *v*/*v*) were employed as mobile phases A and B, respectively. The flow rate was adjusted to 0.3 mL/min. The bile acids were eluted under gradient conditions. The proportion of mobile phase B was maintained at 40% from 0 to 10 min, increased from 40% to 80% from 10 to 23 min, and maintained at 80% from 23 to 28 min. A YMC Triart C18 guard column (2.1 mm I.D. × 5 mm, 1.9 µm, YMC, Kyoto, Japan) and a YMC Triart C18 analytical column (2.1 mm I.D. × 150 mm, 1.9 µm, YMC) were connected and employed at a temperature of 40 °C [45].

The curtain gas, collision gas, ion source gas 1, ion source gas 2, ion spray voltage, and ion source temperature were set to 20 psi, 12 psi, 60 psi, 60 psi, −4500 V, and 300 °C, respectively. Bile acids were analyzed under previously established selected reaction monitoring conditions, as summarized in Table S1. Data acquisition and integration were conducted using the Analyst software version 1.6.2 (SCIEX) and MultiQuant version 2.1.1 (SCIEX).

A calibration curve was constructed within the range of 1–3000 nmol/L. The resulting calibration curves are listed in Table S2.

### 2.4. Statistical Analyses and the Development of Diagnostic Model

**Figure 1:**
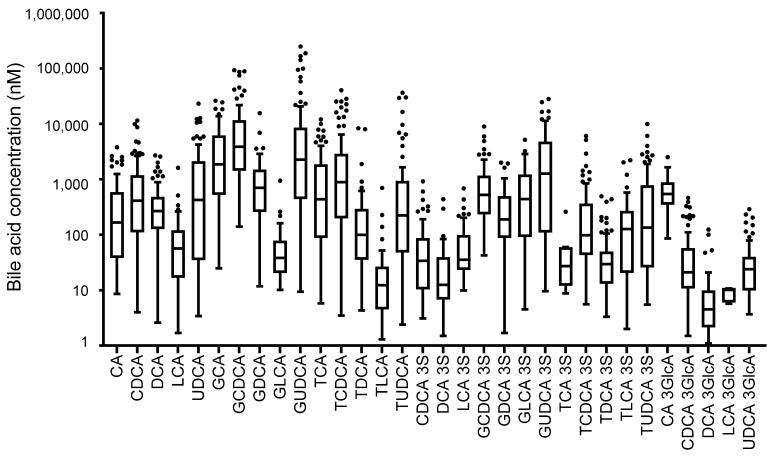
Concentrations of plasma bile acids in all subjects.

GraphPad Prism version 9.3.1 (Dotmatics, Boston, MA, USA) was used to draw Figure 1. JMP Pro version 17.1 (SAS Institute, Cary, NC, USA) was used for all statistical analyses. A nonparametric method was employed for analyzing significant differences for items that did not meet the normal distribution. Diagnostic performance was evaluated using the logistic regression and receiver operating characteristic (ROC) analyses. The area under the curve (AUC) of the ROC curve and its 95% confidence interval (CI) were calculated using GraphPad Prism version 9.3.1.

A stepwise method was used to construct a diagnostic model that integrated multiple test values, including plasma bile acid levels. The threshold *p*-value was set to 0.25 for inclusion and 0.10 for exclusion of selected items to construct the model formula. Targeted items for selection were including clinical test values, each concentration of 32 bile acids, total bile acids (TBA, the sum of 32 bile acids concentrations), and bile acid fractions based on their classification listed in Table S3b. By selecting the items and building a model, the following formula was constructed:

Possibility[4_HCC] = −9.988 − (0.0005504 × GCA [nM]) + (0.04487 × GGT [U/L]) + (0.02604 × GCDCA 3S [nM]) + (0.03243 × GLCA 3S [nM]) + (0.02491 × GUDCA 3S [nM]) − (0.2112 × ALT [U/L]) + (7.833 × ALB [g/dL]) + (0.0006565 × Unconjugates [nM]) − (0.02653 × sulfates [glycine] [nM])(1)

## 3. Results and Discussion

### 3.1. Analysis of Serum Bile Acids in Patients with Hepatocellular Carcinoma and Other Liver Diseases

The patients included in this study are listed in Table 1. No significant differences were observed between sexes. However, significant differences were observed in age, with the age of patients with HCC being significantly higher than that of the other groups. The underlying diseases in the hepatitis, HC, and HCC groups are shown in Table 1, and significant differences were observed in their profiles. Significant differences were observed in AST and ALT levels, whereas no significant differences were found in GGT levels. In chronic hepatitis, AST and ALT levels may return to normal; however, if inflammation persists, a slight increase may be observed. ALT is specific to the liver and is an important indicator of hepatitis [57]. As HC progresses, AST and ALT levels may increase; however, after progression, these levels may return to the normal range, with AST levels often higher than ALT levels [58,59]. The presence of HCC did not necessarily result in increased AST or ALT levels. Increased GGT levels were particularly noticeable in patients with alcoholic hepatitis and alcoholic HC. In cases of HCC, GGT may also be increased; however, if combined with other liver diseases, GGT may also be affected [60]. ALB is a major serum protein synthesized in the liver. It maintains the osmotic pressure of blood and is involved in the transport of nutrients and hormones. However, as HC progresses, the liver’s ability to synthesize ALB decreases, resulting in decreased albumin levels [61]. ChE breaks down acetylcholine and is found in large amounts in the liver, pancreas, and red blood cells. Low ChE levels may indicate impaired liver or pancreatic functions [62,63,64]. There were no significant differences in SCr levels concerning kidney function. UDCA has been approved for the treatment of chronic liver disease and other conditions. There was no significant difference in the rate of UDCA use, which is believed to affect bile acid metabolism. AFP levels were increased in the HCC and TM+ groups, with no significant overall difference observed. This trend was the same as that observed in PIVKA-II and AFP-L3 cells.

All the bile acids were successfully separated and detected (Figure S2). The ranges of plasma bile acid concentrations are shown in Figure 1 and Table S4. This investigation revealed considerable discrepancies in the concentration of bile acids in the blood, which might be due to the body habitus, dietary habits, etiology, disease stage, or other plasma components, such as cholesterol and bilirubin [57,58]. Additionally, variations in the concentrations of different bile acids were determined. The ten bile acids that showed significant differences in ANOVA were UDCA, GCA, GCDCA, GUDCA, TCDCA, GCDCA 3S, GDCA 3S, GUDCA 3S, CDCA 3GlcA, and DCA 3GlcA (Table 2). Significant differences were also observed in the bile acid characteristics in this study, including sulfate and glucuronide conjugates.

### 3.2. Diagnostic Screening Performance Evaluation of Bile Acids as Biomarker Candidates for HCC by Integrating with Conventional Biomarkers

Next, we analyzed the diagnostic performance of HCC using ROC analysis. The initial objective was to identify potential biomarker molecules that could differentiate HC from HCC. Plasma bile acids, other biochemical tests, and TM results are shown in Table S3. Combined bile acid levels were also calculated.

The bile acids and fractions that showed significant differences between HC and HCC were GCA, TCDCA, GCDCA 3S, total bile acids, total primary bile acids (primary bile acids), total conjugated bile acids (conjugates [total]), total glycine conjugated bile acids (glycine), total taurine conjugated bile acids (taurine), total glycine sulfate conjugated bile acids (3-sulfate [glycine]), total taurine sulfate conjugated bile acids (3-sulfate [taurine]), total sulfated bile acids (3-sulfate [total]), total glycine conjugates and glycine conjugated 3-sulfates (glycine [total]), total taurine conjugates and taurine conjugated 3-sulfates (taurine [total]), respectively.

**Figure 2:**
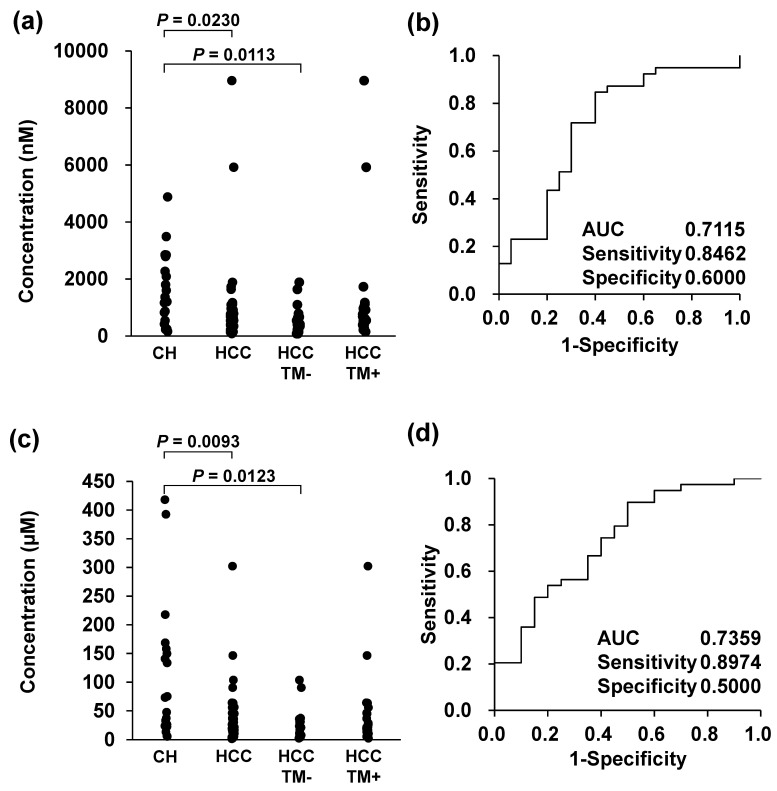
Diagnostic performance evaluation of glycine conjugated chenodeoxycholic acid 3-sulfate (**a**,**b**) and total conjugated bile acids between patients (**c**,**d**) with hepatic cirrhosis and hepatocellular carcinoma (**a**,**c**) plasma concentration and (**b**,**d**) receiver operating characteristic analysis.

GCDCA 3S exhibited the best performance in terms of single-molecule diagnostic performance. Plasma GCDCA 3S levels were significantly lower in patients with HCC than in patients with HC (Figure 2a). The results of the ROC analysis indicated that the AUC for GCDCA 3S was 0.7115 (95% confidence interval (CI), 0.5636–0.8595) (Figure 2b). Furthermore, the total fraction of all conjugated bile acids demonstrated good screening performance (AUC = 0.7359 (95% CI, 0.5992–0.8726), Figure 2d). Previous studies have demonstrated that the concentration of bile acids in the blood of patients with HCC is significantly lower than that in patients with HC [53,59]. Both aforementioned studies reported a reduction in GCDCA levels [53,59]. The observed decline in these metabolites is believed to reflect alterations in the metabolic pathways that occur during HCC progression. [60] A previous study suggested that the reduction in plasma bile acids may be due to decreased excretion from the liver and that toxic and carcinogenic bile acids were accumulated in the liver. Chronic inflammation is one of the factors for HCC development. Alonso-Peña et al. reported that inflammatory cytokines downregulate CYP7A1, responsible for bile acid synthesis, and BAAT, responsible for bile acid amino conjugation [61]. In terms of single bile acids, GCA, TCDCA, and GCDCA 3S showed significantly low concentrations in HCC patients compared with HC patients, which were all conjugated primary bile acids. Thus, the lower concentration of GCA and TCDCA in HCC than in HC is probably due to the downregulation of these enzymes caused by inflammation, and the reduction of GCDCA 3S level might result from GCDCA downregulation. In addition, GCA was reported to attenuate the inflammation by inducing the farnesoid X receptor expression [62]. TCDCA is known to be anti-inflammatory and promote apoptosis [63,64], and GCDCA promotes the apoptosis of normal hepatocytes but cell proliferation of HCC cells [65]. Thus, GCA reduction in HCC might result in promoting inflammation. On the other hand, a reduction in TCDCA and GCDCA 3S is likely to result from the protective reaction. In recent years, several HCC biomarker candidates, such as midkine and osteopontin, have been reported [66]. Midkine is a molecule with excellent biomarker performance and is believed to be produced by tumor cells [67]. Osteopontin is a glycoprotein biosynthesized by various cell types and malignant tissues, with increased levels in patients with HCC [68,69,70]. Bile acids differ from these existing biomarkers and are thought to show a decrease in HCC. Some lipids that compose lipoprotein, other biomarker candidates, were also reported to decrease in HCC [71,72]. Because bile acids are involved in lipid metabolism, it is suggested that they are interrelated in developing HCC. A comparison was conducted between patients with TM− HCC and those with HC (Figure 2a,c). In instances of TM− HCC, the levels of bile acids were slightly diminished compared to those of TM+ HCC (Figure 2a). A positive correlation was identified between the concentration of TM (AFP and PIVKA-II) and several bile acids (CDCA 3S, GCDCA 3S, TCA 3S, 3-sulfate free fraction, and 3-glucuronide fraction), indicating the potential correlation between TM synthesis and bile acid production capacity (Table S5).

**Figure 3:**
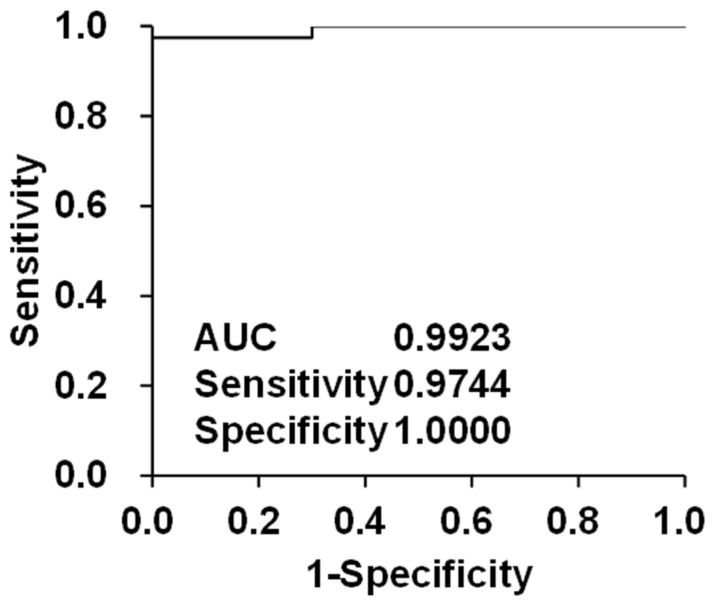
Diagnostic performance evaluation of the integrated diagnostic model between patients with hepatic cirrhosis and hepatocellular carcinoma.

Our objective was to construct a multiparameter discrimination model for HC and HCC to develop a diagnostic screening model. A stepwise method was used to construct an HCC prediction model derived from the entire dataset. Items with a considerable number of missing values were excluded from the analysis. Specifically, PIVKA-II and AFP-L3 were excluded because of the absence of data from patients with HC. Items were incorporated into the model in descending order of *p*-values, with the removal of items when the *p*-value exceeded 0.10, to construct the model. The nine selected items were GGT, ALT, ALB, GCA, GCDCA 3S, GLCA 3S, GUDCA 3S, unconjugates (total unconjugated bile acids: CA, CDCA, DCA, LCA, and UDCA), and 3-sulfate (glycine) (total glycine sulfate conjugated bile acids: GCDCA 3S, GDCA 3S, GLCA 3S, and GUDCA 3S). These markers of liver damage (GGT and ALT), liver function (ALB), primary bile acid conjugates (GCA, GCDCA 3S), secondary bile acid conjugates (GLCA 3S, GUDCA 3S), and the sum of bile acids (unconjugated bile acids and glycine-conjugated bile acid 3-sulfates) were used to construct the model, represented by Equation (1). The likelihood ratio test yielded a *p*-value of less than 0.05 for all nine factors. If the value was positive, it was determined to be HCC; conversely, if it was negative, it was determined to be HC. The results demonstrated a remarkably high AUC (0.9923; 95% CI, 0.9758–1.0000), sensitivity (97.44%), and specificity (100%), suggesting that this model may prove valuable for the prediction of HCC (Figure 3). The model demonstrated the capacity to accurately detect HCC irrespective of the value of the TMs. It is hypothesized that this model integrates changes in hepatic metabolic capacity with changes in the tumor-producing capacity of tumor cells. As there is no precedent for combining multiple bile acids with conventional biochemical tests or for accurately detecting tumors with high precision [47], this approach is regarded as highly novel. ALB is also employed in the Child–Pugh classification, and the range of values observed for HCC was nearly identical to that documented in a previous study [73,74,75]. ALB exhibits a high degree of homology with AFP and is postulated to be associated with HCC, as evidenced by its strong correlation [76]. GGT levels are increased in patients with HC [77,78,79]. In the present study, TM was excluded from the analysis owing to missing data (PIVKA-II and AFP-L3) or lack of statistical significance (AFP). A limitation of this study is the limited number of cases and retrospective cohort analysis. However, further verification is necessary. In addition, we also investigated the performance of the model without bile acids; the diagnostic performance was significantly lower than Equation (1). Therefore, we think that bile acids are important for HCC prediction.

## 4. Conclusions

This study investigated the potential utility of bile acids as biomarkers for HCC. A comprehensive analysis of 32 plasma bile acids was conducted using liquid chromatography/tandem mass spectrometry. Samples from patients with chronic hepatitis, HC, and HCC were analyzed. Initially, we examined the differences in bile acid levels between individuals with HC and HCC identifying significant variations in several bile acids, such as GCA, TCDCA, GCDCA 3S, and some fractions, which might be due to the alteration of the metabolism pathway during HCC development. Subsequently, our aim was to develop a precise method to distinguish patients with HC from those with HCC. We constructed a novel HCC screening tool integrating bile acids with conventional biochemical tests, demonstrating high accuracy. Moving forward, we plan to evaluate the diagnostic efficacy of this model through a comprehensive analysis of a larger patient cohort and to investigate the underlying molecular mechanisms supporting its utility.

## Figures and Tables

**Table 1:** Patient characteristics.

	Chronic Hepatitis (*n* = 20)	Hepatic Cirrhosis (*n* = 20)	HCC			*p* Value
			Total (*n* = 39)	TM− (*n* = 19)	TM+ (*n* = 20)	
Sex, *n* (male/female)	10/10	12/8	24/15	12/7	12/8	0.6976 ^a^
Age, median (IQR)	56 (48.5–65)	53.5 (49.75–62.75)	70 (62–76)	68 (60–76)	70.5 (64.5–78.25)	<0.0001 ^b^
Primary disease, *n* (%)						0.0122 ^b^
HBV	7 (35)	4 (20)	6 (15)	3 (16)	3 (15)	
HCV	5 (25)	4 (20)	20 (51)	12 (63)	8 (40)	
NASH	3 (15)	3 (15)	3 (8)	1 (5)	2 (10)	
PBC	3 (15)	5 (20)	0 (0)	0 (0)	0 (0)	
Alcohol	1 (5)	4 (20)	8 (21)	3 (16)	5 (25)	
Others	AIH, 2 (10) Unknown, 1 (5)	Wilson disease, 1 (5) Unknown, 1 (5)	Unknown, 2 (5)	0 (0)	Unknown, 2 (10)	
GGT, median (IQR) (U/L)	62 (41.25–116.5)	35 (18.25–158.75)	37 (20–85)	25 (18–42)	83.5 (33.25–138.5)	0.1756 ^a^
AST, median (IQR) (U/L)	49.5 (27.5–79)	49 (34.25–62.25)	35 (26–50)	28 (18–42)	45.5 (30.25–56.25)	0.0401 ^a^
ALT, median (IQR) (U/L)	62 (29–99.75)	33 (15.5–50)	28 (17–35)	20 (16–31)	30.5 (17.5–47.25)	0.0005 ^a^
Albumin, median (IQR) (g/dL)	4 (3.7–4.3)	2.95 (1.95–3.425)	3.5 (3.1–3.7)	3.6 (3.4–4.2)	3.3 (3–3.6)	<0.0001 ^a^
ChE, median (IQR) (U/L)	288 (238.75–335.5)	144 (54.5–192.5)	199 (142–253)	221 (176–323)	175 (136–220.5)	<0.0001 ^a^
Scr, median (IQR) (mg/dL)	0.675 (0.58–0.8375)	0.765 (0.5825–1.07)	0.73 (0.64–0.93)	0.77 (0.64–1.06)	0.73 (0.635–0.815)	0.6146 ^a^
UDCA take, *n* (Yes/No)	8/12	11/9	16/23	7/12	9/11	0.5601 ^b^
AFP, median (IQR) (ng/mL) ^c^	-	4.1 (2.425–14.4)	5.4 (3.2–45.8)	4.4 (3.1–5.4)	37.7 (5.175–1570.975)	0.1811 ^a^
PIVKA-II, median (IQR) (mAU/mL) ^d^	-	59.5 (37.5–457.5)	23 (18–134)	19 (16–21)	122.5 (45.25–1749.25)	0.1560 ^a^
AFP-L3, median (IQR) (%) ^e^	-	0.5 (0.5–12.45)	0.5 (0.5–28.2)	0.5 (0.5–0.5)	21.3 (1.575–43.75)	0.8027 ^a^

^a^ Kruskal–Wallis test was performed for significant difference analysis. ^b^ Fisher’s exact test was performed for significant difference analysis. ^c^ *n* = 20 for liver cirrhosis, *n* = 19 for TM−, *n* = 20 for TM+. ^d^ *n* = 16 for liver cirrhosis, *n* = 19 for TM−, *n* = 20 for TM+. ^e^ *n* = 15 for liver cirrhosis, *n* = 19 for TM−, *n* = 20 for TM+. AFP, α-fetoprotein; AIH, autoimmune hepatitis; ALT, alanine aminotransferase; AST, aspartate aminotransferase; ChE, cholinesterase; GGT, γ-glutamyl transferase; HBV, hepatitis B virus; HCC, hepatocellular carcinoma; HCV, hepatitis C virus; IQR, interquartile range; NASH, non-alcoholic steatohepatitis; PBC, primary biliary cholangitis; PIVKA-II, protein induced by vitamin K absence or antagonist-II; Scr, serum creatinine; UDCA, ursodeoxycholic acid.

**Table 2:** Plasma concentrations of bile acids in each sample group.

	Chronic Hepatitis	HC	HCC			*p* Value	
			Total	TM−	TM+	All Group ^b^	CH vs. HCC ^a^
CA (nM) [median (IQR)]	39.0 (27.2–173.1)	189.6 (64.4–619.8)	314.3 (41.5–909.1)	136.8 (37.8–590.1)	426.8 (87.6–1116.9)	0.0721	0.9666
CDCA (nM) [median (IQR)]	127.1 (47.4–452.7)	572.6 (284.8–2044.1)	634.7 (197.1–1723.6)	405.2 (109.9–1532.8)	741.5 (362.6–2504.1)	0.0884	0.9249
DCA (nM) [median (IQR)]	226.4 (126.3–459.7)	358.9 (208.5–1024.1)	259.7 (106.8–407.4)	293.9 (192.3–613.3)	259.7 (37.6–384.4)	0.3202	0.3450
LCA (nM) [median (IQR)]	23.2 (14.8–60.6)	72.7 (22.6–120.0)	103.6 (15.3–154.3)	80.1 (15.3–125.9)	135.8 (16.2–258.3)	0.2012	0.7907
UDCA (nM) [median (IQR)]	135.5 (9.2–998.5)	492.2 (35.8–5296.0)	572.2 (39.3–2440.0)	272.7 (21.3–1971.0)	1327.5 (121.5–3178.2)	0.0242	0.9528
GCA (nM) [median (IQR)]	842.6 (309.4–1845.4)	6060.0 (1952.4–12,455.0)	1725.5 (524.5–5480.0)	1104.6 (283.5–2796.0)	2515.7 (1005.0–7615.3)	0.0008	0.0327
GCDCA (nM) [median (IQR)]	1837.4 (601.6–3608.0)	15,534.5 (2147.6–41,090.0)	5135.0 (1706.2–9624.0)	2896.6 (1201.3–5881.0)	6549.0 (2111.5–12,073.5)	< 0.0001	0.0561
GDCA (nM) [median (IQR)]	622.7 (232.5–1199.6)	750.3 (261.2–1913.4)	755.3 (255.8–1574.6)	836.2 (257.9–1531.3)	379.3 (110.0–2738.4)	0.3466	0.9818
GLCA (nM) [median (IQR)]	21.5 (18.7–30.9)	37.9 (21.4–59.0)	66.6 (21.9–119.6)	68.6 (11.7–127.1)	58.0 (22.7–106.9)	0.1346	0.5084
GUDCA (nM) [median (IQR)]	1201.2 (248.4–4011.0)	2681.5 (824.3–67,072.5)	2281.0 (432.7–11,582.0)	1306.5 (66.9–13985.0)	2666.2 (599.4–8557.0)	0.0158	0.5561
TCA (nM) [median (IQR)]	136.7 (54.8–698.6)	1626.2 (375.0–2698.5)	338.6 (57.2–1756.0)	121.9 (56.6–1145.5)	475.4 (137.1–2404.8)	0.1248	0.0607
TCDCA (nM) [median (IQR)]	391.9 (106.0–1222.5)	3880.0 (578.4–15,372.5)	1126.0 (196.0–2716.9)	437.6 (96.2–2585.5)	1288.5 (263.9–2844.9)	0.0040	0.0313
TDCA (nM) [median (IQR)]	166.0 (49.6–254.8)	77.2 (27.3–380.8)	77.5 (21.3–293.9)	164.4 (48.2–333.5)	56.0 (6.2–160.6)	0.5894	0.9557
TLCA (nM) [median (IQR)]	5.5 (3.8–24.7)	8.3 (5.2–20.8)	18.7 (7.5–35.1)	23.6 (10.4–35.0)	8.5 (3.3–52.1)	0.3826	0.3604
TUDCA (nM) [median (IQR)]	74.6 (19.1–222.2)	473.8 (139.4–6565.0)	241.6 (40.3–931.3)	363.2 (15.0–1171.5)	241.6 (48.4–927.2)	0.0681	0.2966
CDCA 3S (nM) [median (IQR)]	9.0 (6.9–18.8)	72.4 (16.7–88.4)	37.5 (10.7–104.5)	55.4 (9.7–87.7)	33.2 (11.0–135.7)	0.2542	0.6424
DCA 3S (nM) [median (IQR)]	10.9 (6.2–14.8)	16.2 (5.8–82.2)	15.1 (7.0–41.5)	15.1 (6.6–80.1)	14.9 (7.8–33.7)	0.2262	0.9244
LCA 3S (nM) [median (IQR)]	23.4 (13.6–36.5)	37.0 (20.9–175.3)	40.3 (24.3–125.2)	56.0 (27.9–196.5)	32.6 (24.3–96.8)	0.2081	0.9420
GCDCA 3S (nM) [median (IQR)]	284.2 (157.1–504.6)	1285.3 (448.6–2660.0)	538.7 (285.2–960.9)	395.0 (193.1–670.1)	649.6 (422.5–1091.1)	0.0266	0.0230
GDCA 3S (nM) [median (IQR)]	224.7 (85.9–524.2)	413.1 (124.5–820.3)	170.2 (81.1–286.7)	165.2 (107.8–409.2)	189.9 (44.5–266.6)	0.0324	0.2418
GLCA 3S (nM) [median (IQR)]	517.0 (205.5–1243.2)	395.9 (59.8–1527.8)	419.0 (91.0–1216.2)	655.0 (326.8–1366.0)	280.1 (15.4–599.7)	0.9949	0.8369
GUDCA 3S (nM) [median (IQR)]	338.0 (54.3–2787.6)	2803.8 (205.9–10,962.5)	1842.0 (110.2–3140.0)	544.0 (60.8–3140.0)	2016.4 (821.9–3192.5)	0.0040	0.3658
TCA 3S (nM) [median (IQR)]	30.0 (24.8–59.7)	14.5 (11.2–60.1)	133.9 (8.8–259.0)	Not detected	133.9 (8.8–259.0)	0.3906	1.0000
TCDCA 3S (nM) [median (IQR)]	52.0 (18.9–109.2)	153.9 (79.2–1074.5)	103.8 (43.9–365.3)	61.1 (27.8–129.7)	189.7 (99.5–385.1)	0.1153	0.3320
TDCA 3S (nM) [median (IQR)]	31.7 (17.6–97.7)	40.1 (13.4–150.2)	23.4 (11.4–35.4)	23.4 (13.4–35.2)	19.2 (6.8–49.4)	0.2611	0.1485
TLCA 3S (nM) [median (IQR)]	155.1 (36.3–310.8)	137.6 (33.2–281.7)	95.9 (17.5–260.7)	151.8 (70.7–297.2)	70.2 (3.1–160.8)	0.7421	0.8452
TUDCA 3S (nM) [median (IQR)]	83.5 (12.1–490.1)	869.0 (25.9–2180.0)	134.8 (39.7–555.4)	91.2 (19.1–545.4)	162.9 (78.1–726.4)	0.1929	0.4887
CA 3GlcA (nM) [median (IQR)]	548.4 (345.4–637.7)	657.7 (252.1–1100.9)	532.5 (377.4–1018.6)	528.7 (260.8–1018.6)	583.3 (382.9–1042.4)	0.1740	0.9423
CDCA 3GlcA (nM) [median (IQR)]	9.8 (3.7–20.7)	37.2 (12.7–139.0)	23.6 (16.4–57.5)	23.6 (16.4–50.8)	23.1 (16.2–73.7)	0.0079	0.7513
DCA 3GlcA (nM) [median (IQR)]	4.0 (2.6–6.2)	10.3 (2.2–52.5)	4.4 (1.9–6.3)	4.6 (1.7–11.5)	4.2 (2.1–5.0)	0.0209	0.3210
LCA 3GlcA (nM) [median (IQR)]	Not detected	10.4 (10.4–10.4)	8.5 (5.9–10.8)	8.6 (6.4–10.8)	8.2 (5.7–10.6)	0.5505	1.0000
UDCA 3GlcA (nM) [median (IQR)]	28.6 (9.3–55.5)	21.7 (7.5–35.7)	25.4 (10.9–42.0)	28.7 (12.9–45.9)	16.5 (9.4–50.9)	0.8427	0.7473

^a^ Steel–Dwass test was performed for significant difference analysis. ^b^ ANOVA was performed for significant difference analysis. 3GlcA, 3-glucuronide; 3S, 3-sulfate; CA, cholic acid; CDCA, chenodeoxycholic acid; DCA, deoxycholic acid; GCA, glycine-conjugated cholic acid; GCDCA, glycine-conjugated chenodeoxycholic acid; GDCA, glycine-conjugated deoxycholic acid; GLCA, glycine-conjugated lithocholic acid; GUDCA, glycine-conjugated ursodeoxycholic acid; IQR, interquartile range; LCA, lithocholic acid; TCA, taurine-conjugated cholic acid; TCDCA, taurine-conjugated chenodeoxycholic acid; TDCA, taurine-conjugated deoxycholic acid; TLCA, taurine-conjugated lithocholic acid; TUDCA, taurine-conjugated ursodeoxycholic acid; UDCA, ursodeoxycholic acid.

## Data Availability

Data are contained within the article or Supplementary Materials.

## Supplementary Materials

The following supporting information can be downloaded at: https://www.mdpi.com/article/10.3390/metabo14090513/s1, Figure S1: The chemical structures of bile acids; Figure S2: SRM chromatograms of bile acids; Table S1: SRM conditions for bile acids and internal standards; Table S2: Calibration curves for bile acids; Table S3: All data of subjects included this study (a) all data (b) Calculation of bile acid fractions; Table S4: Concentration range of plasma bile acids; Table S5: Correlation of all items.
