# Supplementary material for: Liquid Chromatography/Tandem Mass Spectrometry-Based Simultaneous Analysis of 32 Bile Acids in Plasma and Conventional Biomarker-Integrated Diagnostic Screening Model Development for Hepatocellular Carcinoma
Source: Metabolites. 2024 Sep 23;14(9):513. doi: 10.3390/metabo14090513 (PMC11433973; doi:10.3390/metabo14090513)
Supplement: Supplementary file 1 [file metabolites-14-00513-s001.zip › FigS_1.0.pptx]

## Slide 1
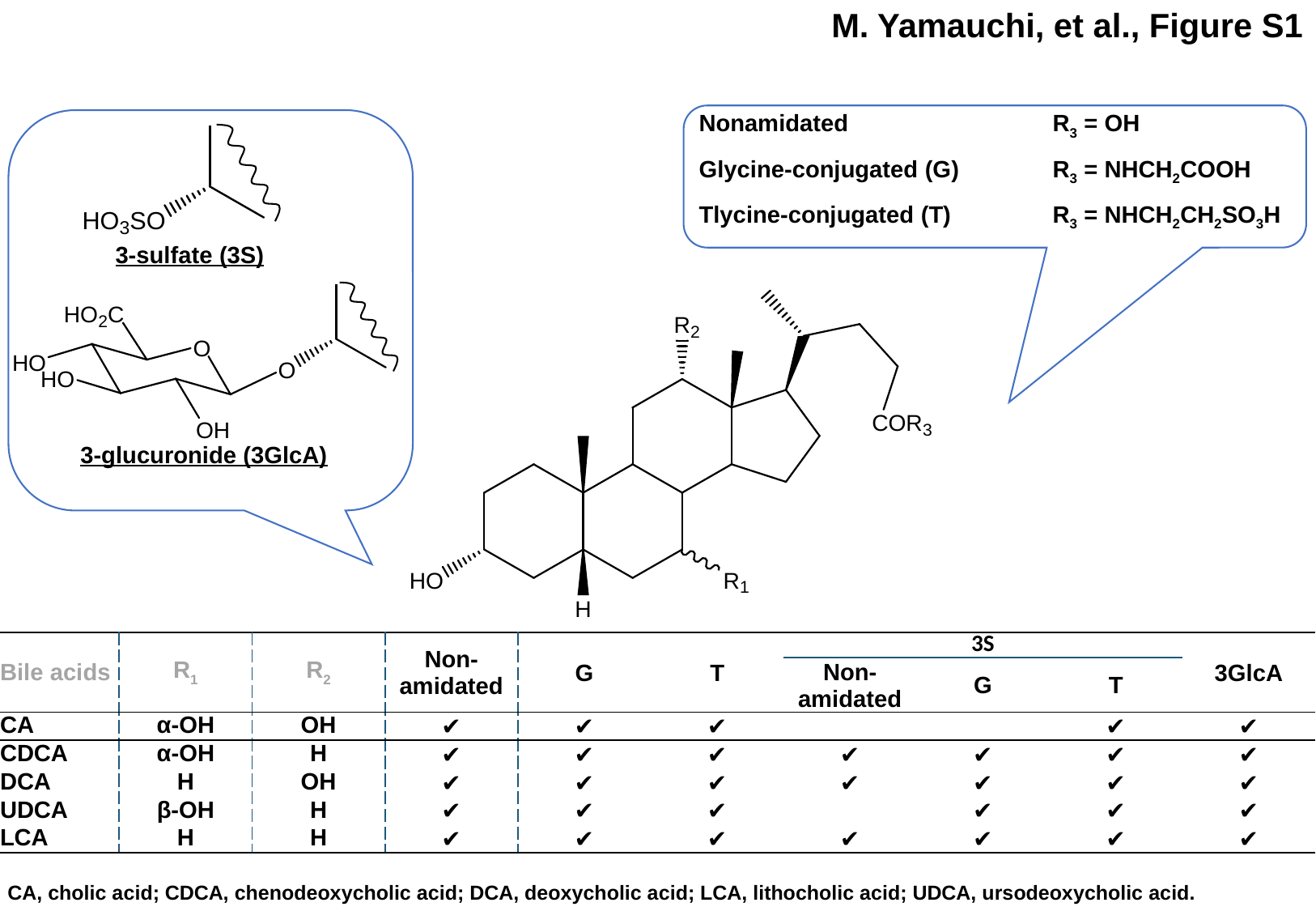

# M. Yamauchi, et al., Figure S1
| Nonamidated | R3 = OH |
| --- | --- |
| Glycine-conjugated (G) | R3 = NHCH2COOH |
| Tlycine-conjugated (T) | R3 = NHCH2CH2SO3H |
3-sulfate (3S)
3-glucuronide (3GlcA)
| Bile acids | R1 | R2 | Non-amidated | G | T | 3S | | | 3GlcA |
| --- | --- | --- | --- | --- | --- | --- | --- | --- | --- |
| | | | | | | Non-amidated | G | T | |
| CA | α-OH | OH | ✔ | ✔ | ✔ | | | ✔ | ✔ |
| CDCA | α-OH | H | ✔ | ✔ | ✔ | ✔ | ✔ | ✔ | ✔ |
| DCA | H | OH | ✔ | ✔ | ✔ | ✔ | ✔ | ✔ | ✔ |
| UDCA | β-OH | H | ✔ | ✔ | ✔ | | ✔ | ✔ | ✔ |
| LCA | H | H | ✔ | ✔ | ✔ | ✔ | ✔ | ✔ | ✔ |
CA, cholic acid; CDCA, chenodeoxycholic acid; DCA, deoxycholic acid; LCA, lithocholic acid; UDCA, ursodeoxycholic acid.

## Slide 2
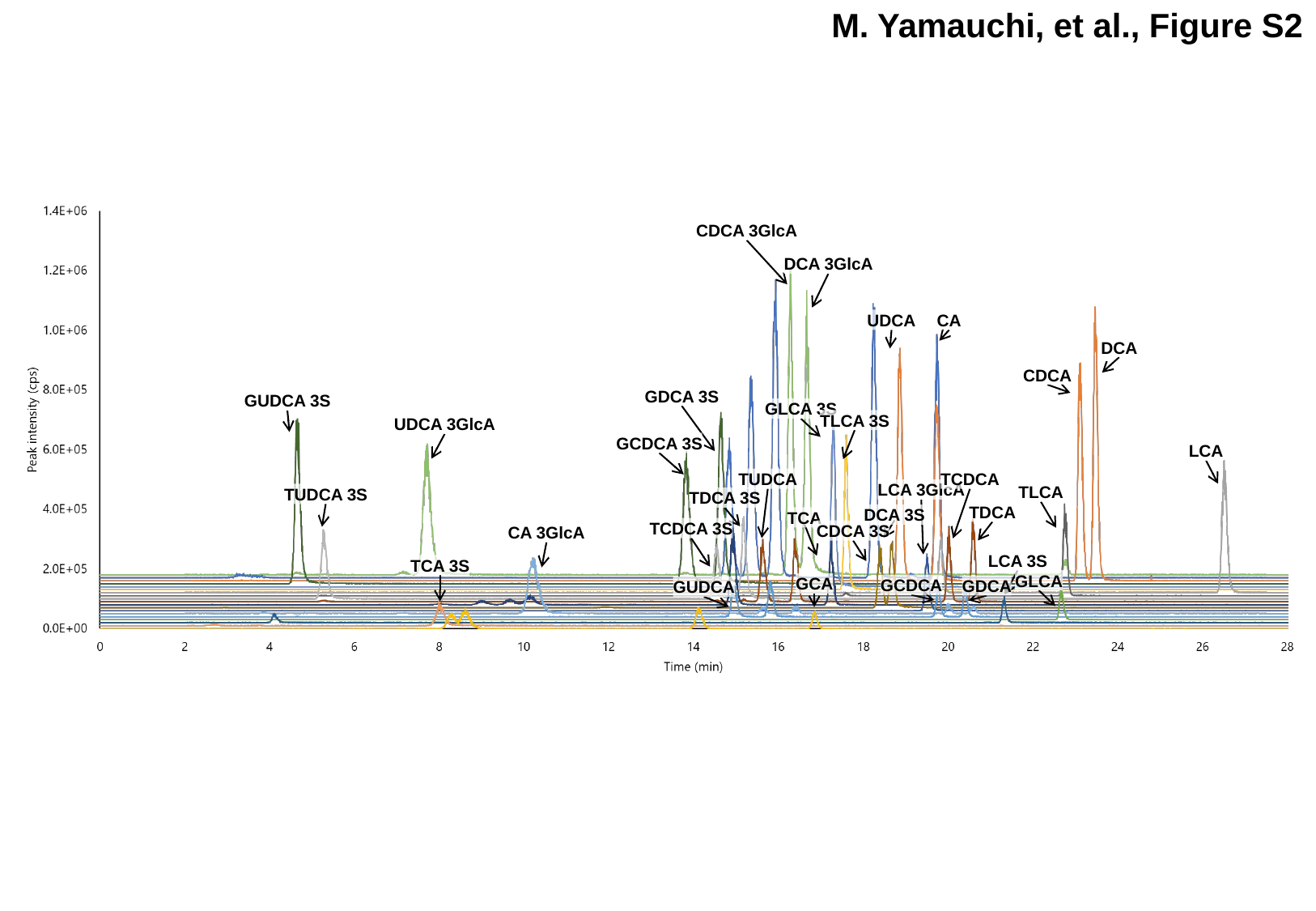

# M. Yamauchi, et al., Figure S2
CDCA 3GlcA
DCA 3GlcA
UDCA
CA
DCA
CDCA
GDCA 3S
GUDCA 3S
GLCA 3S
TLCA 3S
UDCA 3GlcA
GCDCA 3S
LCA
TUDCA
TCDCA
LCA 3GlcA
TLCA
TUDCA 3S
TDCA 3S
TDCA
DCA 3S
TCA
TCDCA 3S
CDCA 3S
CA 3GlcA
LCA 3S
TCA 3S
GLCA
GCA
GCDCA
GDCA
GUDCA
