# Supplementary material for: Liquid Chromatography/Tandem Mass Spectrometry-Based Simultaneous Analysis of 32 Bile Acids in Plasma and Conventional Biomarker-Integrated Diagnostic Screening Model Development for Hepatocellular Carcinoma
Source: Metabolites. 2024 Sep 23;14(9):513. doi: 10.3390/metabo14090513 (PMC11433973; doi:10.3390/metabo14090513)
Supplement: Supplementary file 1 [file metabolites-14-00513-s001.zip › Table S1_1.0.pdf]

Table S1. SRM conditions for bile acids and internal standards.

Number	Analyte	SRM transitions	DP (V)	CE (V)	CXP (V)	EP (V)
1	CA	*m/z* 453 → 407	30	25	15	6
2	CDCA	*m/z* 437 → 391	25	25	15	6
3	DCA	*m/z* 437 → 391	25	25	15	6
4	LCA	*m/z* 421 → 375	25	20	15	6
5	UDCA	*m/z* 437 → 391	25	25	15	6
6	GCA	*m/z* 464 → 74	120	80	10	6
7	GCDCA	*m/z* 448 → 74	120	80	10	6
8	GDCA	*m/z* 448 → 74	120	80	10	6
9	GLCA	*m/z* 432 → 74	120	80	10	6
10	GUDCA	*m/z* 448 → 74	120	80	10	6
11	TCA	*m/z* 514 → 80	100	150	11	8
12	TCDCA	*m/z* 498 → 80	100	130	11	8
13	TDCA	*m/z* 498 → 80	100	130	11	8
14	TLCA	*m/z* 482 → 80	100	130	11	8
15	TUDCA	*m/z* 498 → 80	100	130	11	8
16	CDCA 3S	*m/z* 471 → 97	45	110	15	6
17	DCA 3S	*m/z* 471 → 97	45	110	15	6
18	LCA 3S	*m/z* 455 → 97	120	110	11	6
19	GCDCA 3S	*m/z* 528 → 448	10	44	21	6
20	GDCA 3S	*m/z* 528 → 448	10	44	21	6
21	GLCA 3S	*m/z* 512 → 432	5	44	5	8
22	GUDCA 3S	*m/z* 528 → 448	10	44	21	6
23	TCA 3S	*m/z* 296 → 496	70	32	11	6
24	TCDCA 3S	*m/z* 289 → 480	70	32	11	6
25	TDCA 3S	*m/z* 289 → 480	70	32	11	6
26	TLCA 3S	*m/z* 280 → 464	20	32	13	8
27	TUDCA 3S	*m/z* 289 → 480	70	32	11	6
28	CA 3GlcA	*m/z* 583 → 583	60	30	13	10
29	CDCA 3GlcA	*m/z* 567 → 567	60	30	13	10
30	DCA 3GlcA	*m/z* 567 → 567	60	30	13	10
31	LCA 3GlcA	*m/z* 551 → 375	16	57	18	10
32	UDCA 3GlcA	*m/z* 567 → 567	60	30	13	10
33	3,7,12-[^{18}O]CA	*m/z* 459 → 413	30	25	15	6

Number	Analyte	SRM transitions	DP (V)	CE (V)	CXP (V)	EP (V)
34	2,2,4,4-[^{2}H]DCA	*m/z* 441 → 395	60	30	13	10
35	2,2,4,4-[^{2}H]LCA	*m/z* 425 → 379	60	30	13	10
36	3,7-[^{18}O]GCA	*m/z* 470 → 74	120	80	10	6
37	3,7-[^{18}O, ^{2}H]GCDCA	*m/z* 454 → 74	120	80	10	6
38	3-[^{18}O, ^{2}H]GLCA	*m/z* 435 → 74	120	80	10	6
39	3,12-[^{18}O, ^{2}H]TDCA	*m/z* 504 → 80	100	130	11	8
40	3-[^{18}O, ^{2}H]TLCA	*m/z* 485 → 80	100	130	11	8
41	S7β-*nor*-Δ^{5}-CA	*m/z* 455 → 97	170	96	13	10
42	E2 3S-[$^{2}H_4$]	*m/z* 355 → 275	155	52	15	10

As some glucuronides (No. 28-30, 32), the selected ion monitoring analyses were performed because were set the same monitoring ions on Q1 and Q3.

CE, Collision energy; CXP, Collision cell exit potential; DP, Declustering potential; EP, Entrance potential; LC, Liquid chromatography; MS/MS, Tandem mass spectrometry; SRM, Selected reaction monitoring.

3GlcA, 3-glucuronide; 3S, 3-sulfate; CA, Cholic acid; CDCA, Chenodeoxycholic acid; DCA, Deoxycholic acid; E2 3S-[$^{2}H_4$], 17β-estradiol-2,4,16,16-[$^{2}H_4$] 3-sulfate; GCA, Glycine-conjugated cholic acid; GCDCA, Glycine-conjugated chenodeoxycholic acid; GDCA, Glycine-conjugated deoxycholic acid; GLCA, Glycine-conjugated lithocholic acid; GUDCA, Glycine-conjugated ursodeoxycholic acid; LCA, Lithocholic acid; S7β-*nor*-Δ^{5}-CA, 3β-sulfooxy-7β-hydroxy-23-*nor*-5-cholenoic acid; TCA, Taurine-conjugated cholic acid; TCDCA, Taurine-conjugated chenodeoxycholic acid; TDCA, Taurine-conjugated deoxycholic acid; TLCA, Taurine-conjugated lithocholic acid; TUDCA, Taurine-conjugated ursodeoxycholic acid; UDCA, Ursodeoxycholic acid.
