# Supplementary material for: Liquid Chromatography/Tandem Mass Spectrometry-Based Simultaneous Analysis of 32 Bile Acids in Plasma and Conventional Biomarker-Integrated Diagnostic Screening Model Development for Hepatocellular Carcinoma
Source: Metabolites. 2024 Sep 23;14(9):513. doi: 10.3390/metabo14090513 (PMC11433973; doi:10.3390/metabo14090513)
Supplement: Supplementary file 1 [file metabolites-14-00513-s001.zip › Table S2_1.0.pdf]

Table S2. Calibration curves for bile acids.

Analytes	IS	Calibration range (nM)	R^2	Retention time (min)
CA	3,7,12-[^{18}O]CA	3-3000	0.994	19.9
CDCA	2,2,4,4-[^{2}H]DCA	1-3000	0.999	23.2
DCA	2,2,4,4-[^{2}H]DCA	3-3000	0.997	23.5
LCA	2,2,4,4-[^{2}H]LCA	1-3000	0.994	26.5
UDCA	2,2,4,4-[^{2}H]DCA	1-3000	0.996	19.0
GCA	3,7-[^{18}O]GCA	3-3000	0.999	16.9
GCDCA	3,7-[^{18}O, ^{2}H]GCDCA	10-3000	0.999	19.8
GDCA	3,7-[^{18}O, ^{2}H]GCDCA	10-3000	0.999	20.4
GLCA	3-[^{18}O, ^{2}H]GLCA	3-1000	1.000	22.7
GUDCA	3,7-[^{18}O, ^{2}H]GCDCA	1-3000	0.971	15.0
TCA	3,12-[^{18}O, ^{2}H]TDCA	1-3000	0.996	17.3
TCDCA	3,12-[^{18}O, ^{2}H]TDCA	1-3000	0.997	20.0
TDCA	3,12-[^{18}O, ^{2}H]TDCA	1-3000	0.999	20.6
TLCA	3-[^{18}O, ^{2}H]TLCA	1-1000	0.998	22.8
TUDCA	3,12-[^{18}O, ^{2}H]TDCA	3-3000	0.999	15.7
CDCA 3S	3,7,12-[^{18}O]CA	1-3000	1.000	18.5
DCA 3S	3,7,12-[^{18}O]CA	1-1000	1.000	18.8
LCA 3S	3,7,12-[^{18}O]CA	3-1000	0.998	21.4
GCDCA 3S	3,7,12-[^{18}O]CA	10-3000	1.000	13.9
GDCA 3S	3,7-[^{18}O]GCA	1-3000	0.999	14.7
GLCA 3S	3,12-[^{18}O, ^{2}H]TDCA	3-3000	0.999	17.3
GUDCA 3S	E2 3S-[$^{2}H_4$]	3-3000	0.993	4.7
TCA 3S	3,7,12-[^{18}O]CA	3-1000	0.999	8.2
TCDCA 3S	3-[^{18}O, ^{2}H]TLCA	1-3000	0.994	14.6
TDCA 3S	3,7,12-[^{18}O]CA	1-1000	0.999	15.3
TLCA 3S	3,7,12-[^{18}O]CA	1-3000	0.998	17.6
TUDCA 3S	E2 3S-[$^{2}H_4$]	1-3000	0.990	5.3
CA 3GlcA	3,7,12-[^{18}O]CA	10-3000	1.000	10.0
CDCA 3GlcA	3,7,12-[^{18}O]CA	1-1000	0.999	16.8
DCA 3GlcA	3,7,12-[^{18}O]CA	1-300	0.997	16.4
LCA 3GlcA	3-[^{18}O, ^{2}H]GLCA	1-300	0.994	19.6
UDCA 3GlcA	S7β-*nor*-Δ^5-CA	3-1000	0.999	8.3

3GlcA, 3-glucuronide; 3S, 3-sulfate; CA, Cholic acid; CDCA, Chenodeoxycholic acid; DCA, Deoxycholic acid; E2 3S-[$^{2}H_4$], 17β-estradiol-2,4,16,16-[$^{2}H_4$] 3-sulfate; GCA, Glycine-conjugated cholic acid; GCDCA, Glycine-conjugated chenodeoxycholic acid; GDCA, Glycine-conjugated deoxycholic acid; GLCA, Glycine-conjugated lithocholic acid; GUDCA, Glycine-conjugated ursodeoxycholic acid; LCA, Lithocholic acid; S7β-*nor*-Δ^5-CA, 3β-sulfooxy-7β-hydroxy-23-*nor*-5-cholenoic acid; TCA, Taurine-conjugated cholic acid; TCDCA, Taurine-conjugated chenodeoxycholic acid; TDCA, Taurine-conjugated deoxycholic acid; TLCA, Taurine-conjugated lithocholic acid; TUDCA, Taurine-conjugated ursodeoxycholic acid; UDCA, Ursodeoxycholic acid.
