# Supplementary material for: Liquid Chromatography/Tandem Mass Spectrometry-Based Simultaneous Analysis of 32 Bile Acids in Plasma and Conventional Biomarker-Integrated Diagnostic Screening Model Development for Hepatocellular Carcinoma
Source: Metabolites. 2024 Sep 23;14(9):513. doi: 10.3390/metabo14090513 (PMC11433973; doi:10.3390/metabo14090513)
Supplement: Supplementary file 1 [file metabolites-14-00513-s001.zip › Table S4_1.0.pdf]

Table S4_1.0.docx

Table S4. Concentration range of plasma bile acids.

Analytes	Concentration (nM) [Median (IQR)]
CA	273.2 (24.2-2028.5)
CDCA	1545.5 (495.2-5789.5)
DCA	2882.3 (1254.4-9655.5)
LCA	731.2 (255.8-1327.3)
UDCA	29.5 (20.5-71.3)
GCA	1548.9 (299.9-7412.0)
GCDCA	337.9 (77.1-1763.4)
GDCA	760.8 (188.5-2872.5)
GLCA	104.3 (34.7-250.8)
GUDCA	11.9 (4.2-26.4)
TCA	175.2 (32.1-935.4)
TCDCA	31.0 (10.2-85.9)
TDCA	11.9 (6.7-37.8)
TLCA	35.0 (23.3-96.2)
TUDCA	273.2 (24.2-2028.5)
CDCA 3S	1545.5 (495.2-5789.5)
DCA 3S	2882.3 (1254.4-9655.5)
LCA 3S	731.2 (255.8-1327.3)
GCDCA 3S	492.0 (215.8-1098.8)
GDCA 3S	184.5 (93.8-503.5)
GLCA 3S	401.8 (92.4-1182.5)
GUDCA 3S	752.1 (84.5-3747.8)
TCA 3S	27.4 (12.0-60.0)
TCDCA 3S	93.0 (37.9-328.6)
TDCA 3S	28.1 (13.5-50.2)
TLCA 3S	97.7 (27.9-262.9)
TUDCA 3S	129.1 (25.3-766.4)
CA 3GlcA	530.6 (280.9-834.6)
CDCA 3GlcA	20.6 (10.4-50.9)
DCA 3GlcA	4.3 (2.1-9.5)
LCA 3GlcA	10.4 (6.1-10.7)
UDCA 3GlcA	23.0 (9.6-38.7)

3GlcA, 3-glucuronide; 3S, 3-sulfate; CA, Cholic acid; CDCA, Chenodeoxycholic acid; DCA,

Deoxycholic acid; E2 3S-[$^{2}H_4$], 17β-estradiol-2,4,16,16-[$^{2}H_4$] 3-sulfate; GCA, Glycine-conjugated cholic acid; GCDCA, Glycine-conjugated chenodeoxycholic acid; GDCA, Glycine-conjugated deoxycholic acid; GLCA, Glycine-conjugated lithocholic acid; GUDCA, Glycine-conjugated ursodeoxycholic acid; IQR, interquartile range; LCA, Lithocholic acid; S7β-*nor*-Δ^5-CA, 3β-sulfooxy-7β-hydroxy-23-*nor*-5-cholenoic acid; TCA, Taurine-conjugated cholic acid; TCDCA, Taurine-conjugated chenodeoxycholic acid; TDCA, Taurine-conjugated deoxycholic acid; TLCA, Taurine-conjugated lithocholic acid; TUDCA, Taurine-conjugated ursodeoxycholic acid; UDCA, Ursodeoxycholic acid.
